# Using Deep Convolutional Neural Networks for Neonatal Brain Image Segmentation

**DOI:** 10.3389/fnins.2020.00207

**Published:** 2020-03-26

**Authors:** Yang Ding, Rolando Acosta, Vicente Enguix, Sabrina Suffren, Janosch Ortmann, David Luck, Jose Dolz, Gregory A. Lodygensky

**Affiliations:** ^1^The Canadian Neonatal Brain Platform (CNBP), Montreal, QC, Canada; ^2^Department of Management and Technology, Université du Québec à Montréal, Montreal, QC, Canada; ^3^Laboratory for Imagery, Vision and Artificial Intelligence (LIVIA), École de Technologie Supérieure, Montreal, QC, Canada; ^4^Department of Pediatrics, Sainte-Justine University Hospital Research Center, Montreal, QC, Canada; ^5^Department of Pharmacology and Physiology, University of Montreal, Montreal, QC, Canada

**Keywords:** neonatal brain, brain segmentation, machine learning (artificial intelligence), convolutional neural network, T2-weighed MRI

## Abstract

**Introduction:**

Deep learning neural networks are especially potent at dealing with structured data, such as images and volumes. Both modified LiviaNET and HyperDense-Net performed well at a prior competition segmenting 6-month-old infant magnetic resonance images, but neonatal cerebral tissue type identification is challenging given its uniquely inverted tissue contrasts. The current study aims to evaluate the two architectures to segment neonatal brain tissue types at term equivalent age.

**Methods:**

Both networks were retrained over 24 pairs of neonatal T1 and T2 data from the Developing Human Connectome Project public data set and validated on another eight pairs against ground truth. We then reported the best-performing model from training and its performance by computing the Dice similarity coefficient (DSC) for each tissue type against eight test subjects.

**Results:**

During the testing phase, among the segmentation approaches tested, the dual-modality HyperDense-Net achieved the best statistically significantly test mean DSC values, obtaining 0.94/0.95/0.92 for the tissue types and took 80 h to train and 10 min to segment, including preprocessing. The single-modality LiviaNET was better at processing T2-weighted images than processing T1-weighted images across all tissue types, achieving mean DSC values of 0.90/0.90/0.88 for gray matter, white matter, and cerebrospinal fluid, respectively, while requiring 30 h to train and 8 min to segment each brain, including preprocessing.

**Discussion:**

Our evaluation demonstrates that both neural networks can segment neonatal brains, achieving previously reported performance. Both networks will be continuously retrained over an increasingly larger repertoire of neonatal brain data and be made available through the Canadian Neonatal Brain Platform to better serve the neonatal brain imaging research community.

## Introduction

The magnetic resonance imaging (MRI) study of brain development since birth represents one of the crucial modern techniques to improve our understanding of developmental neuroscience and help identify the long-term links between brain injuries and respective developmental consequences. However, despite mature analytical methods to process adult human brain MRIs, analyses of brains during development and especially at the neonatal stage remain difficult as a result of isolated tools development and difficulty with data acquisition. The most important step before performing quantitative brain analyses is the tissue class segmentation of the brain. Neonatal brain medical imaging tissue type identification is especially challenging given its typically inverted T1/T2 tissue contrast compared to adults (Shroff et al., 2010). Moreover, the amount of high-quality public neonatal research neural MRI data sets is far rarer in comparison to adult neural MRI data, making training, development, and adoption of neonatal-specific brain segmentation approaches challenging. From our experience in attempting to implement majority of the open-source neonatal segmentation approaches at the Canadian Neonatal Brain Platform (CNBP)^1^, many existing computer-vision-based solutions failed to generalize beyond the respective niche of privately held training data set. As part of our organizational mandates, CNBP aims to validate and provide a large variety of neonatal brain MRI processing approaches. In this article, we focused primarily on public-data-based open-source deep learning approaches in the context of neonatal brain tissue segmentation.

Recent years have witnessed an explosive growth in the number of deep learning methods – especially convolutional neural network (CNNs) – for many vision problems, such as classification (Krizhevsky et al., 2012), detection (Ren et al., 2015), and semantic segmentation (Long et al., 2015). These models are capable of learning highly complex patterns by stacking multiple layers of convolutions and non-linear operations, presenting impressive capabilities to learn abstract representations from raw structured data in a data-driven manner. Particularly, the medical field has greatly benefited from these deep models, which have become the *de facto* solution for many of these tasks in highly important fields, such as radiology, oncology, or neuroimaging (Litjens et al., 2017).

Despite the fast adoption of these models in medical imaging, there have been relatively few large-scale efforts to find the top performer in pediatric brain segmentation using standardized open data sets (Akkus et al., 2017). Two particularly large-scale relevant competitions are known to date: the 2012 Neonatal Brains Segmentation Challenge^2^ and the 2017 iSeg 6-month Infant Brain Magnetic Resonance Imaging Segmentation Challenge^3^, both hosted as part of the respective Medical Image Computing and Computer Assisted Intervention Society (MICCAI) conferences. Out of the two competitions, the 2017 competition was particularly relevant as most contestants used derivation of CNN architecture forgoing traditional computer vision techniques, and some top performers openly shared their network architecture designs and code bases.

Outside of the iSeg 2017 competition and its related publications (Wang et al., 2019), which focus on 6-month-old infants, there have been few other proposed deep-learning-based segmentation approaches in neonates, despite numerous applications in either older infants (Zhang et al., 2015) or adults (Chen et al., 2018). The only applied neural network approach to solve neonatal tissue segmentation to date is from Moeskops et al. (2016). They proposed an integrated segmentation pipeline that reportedly can handle data from neonates all the way to 70-year-old adults using mini-patch-based 2D convolution approaches while only requiring a single anatomical reference MRI to achieve a respectable Dice score of at least 0.8 across five different data sets.

The objective of the current study is to evaluate both LiviaNET (Dolz et al., 2018b) and HyperDense-Net (Dolz et al., 2018a) architectures for neonatal brain imaging data. While both networks have demonstrated good performance on relevant tasks, such as in subcortical brain segmentation and in 6-month-old infant brain imaging data with diminished T1/T2 contrasts (Wang et al., 2012), their performance on neonatal-specific data remains untested. We hypothesize with a high-quality data set and ground truth, such as those from the publicly available Developing Human Connectome Project (DHCP) first-release neonatal data set (Hughes et al., 2017), we can achieve performance comparable to what prior modified LiviaNET and HyperDense-Net achieved in the adult and 6-month-old infant brain challenges. We aim to retrain both networks using the DHCP data set to validate the generalizability and the suitability of these network architectures in segmenting MRI brain tissue classes of neonatal brain images.

## Methods

### Experimental Data: Participants

The participants were infants born at term from the publicly available DHCP by Hughes et al. (2017). DHCP is the first open-access data release of brain images of 40 healthy neonates born at term who had an MRI shortly after birth (37–44 weeks of gestational age). With these data, we had access to both raw data and tissue segmentation ground truth, generated using DrawEM and complemented further via manual correction, for training and validations. Additional MRI data-acquisition-related information is included in Supplementary Method as well as the original publication.

### Experimental Data: Preprocessing

**FIGURE 1:**
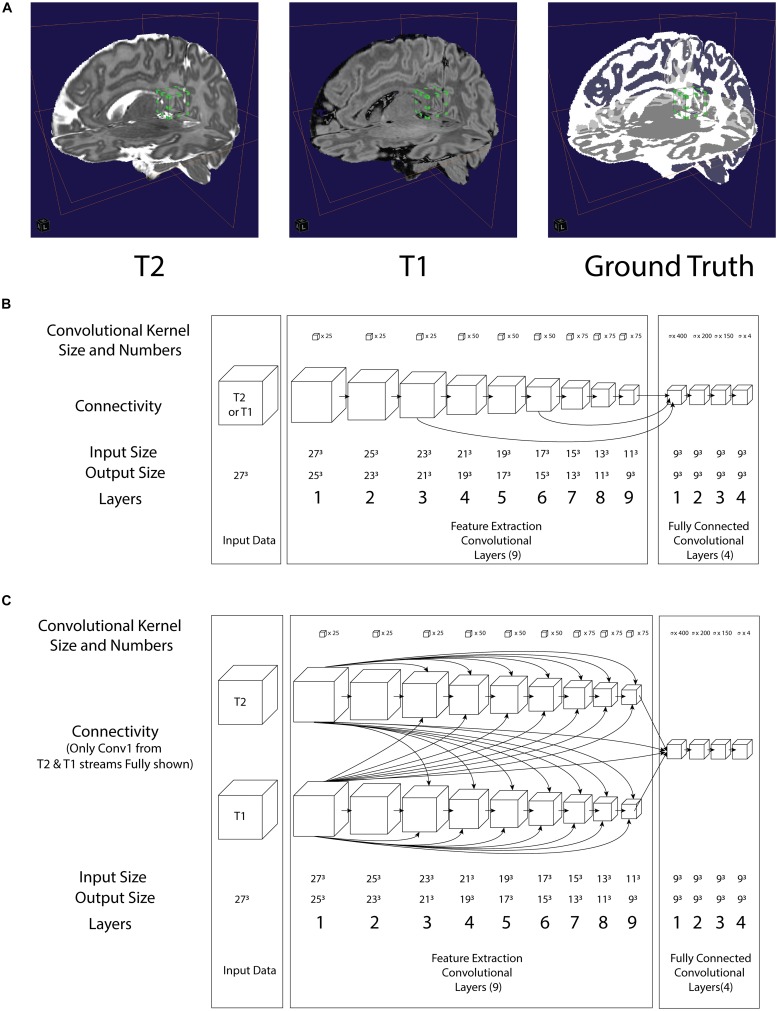
**(A)** Illustration of a 3D convolution regional input (27 pixels^3^) to both neural networks in relation to T2, T1 and Ground truth. **Bounding box with green tab:** input volume to the network **(B)** architecture of the LiviaNET illustrating major layer wise connections along with key parameters **(C)** architecture of HyperDense-Net neural network architecture including key parameters.

The training input was preprocessed based on the source image provided as part of the DHCP data made available (Hughes et al., 2017), namely, magnetic resonance bias-field correction with the N4 algorithm (Tustison et al., 2010) as implemented in Slicer 4.10.1 on our computational platform (see **Implementation: Computation Platform** section), launched with the command “Slicer – launch N4ITKBiasFieldCorrection.” Then the brain was extracted using the Brain Extraction Tool (BET2) with the default options (i.e., no additional customized command flags) from FMRIB Software Library (Smith, 2002; see Figure 1A). All T1-weighted images have been co-registered to the T2-weighted volumes using rigid alignment as implemented in SPM12 (Ashburner et al., 2014) in MATLAB (R2017b) (MathWorks Inc., Natick, MA, United States) running on our computational platform.

### Experimental Data: Ground Truth Segmentation

As part of the DHCP data release, these neonatal brain MRIs were already segmented using the DHCP data pipeline built using the DrawEM module from the Medical Image Registration ToolKit (MIRTK) tool package (Makropoulos et al., 2014). DrawEM is an atlas-based segmentation technique that segments the volumes into 87 regions. Manually labeled atlases, annotated by an expert neuroanatomist (Gousias et al., 2012), were registered to the volume, and their labels were fused to the subject space to provide structure priors. Segmentation was then performed with an expectation–maximization scheme that combines the structure priors and an intensity model of the volume. The 87 regions were further merged to provide nine tissue segmentation labels provided with the DHCP release: (1) cerebrospinal fluid (CSF), (2) cortical gray matter (GM), (3) white matter (WM), (4) background, (5) ventricles, (6) cerebellum, (7) deep GM, (8) brain stem, and (9) hippocampus and amygdala. Since both LiviaNET and HyperDense-Net demonstrated their respective previous performance when dealing with four class labels (i.e., GM, WM, CSF, and others), we used the image calculator (ImCalc) function of SPM12 implemented in MATLAB R (2017b) (MathWorks Inc., Natick, MA, United States) to combine the existing nine DHCP class labels into the desired classes. More specifically, we combined together the cortical GM, cerebellum, deep GM, brainstem, and hippocampus and amygdala into the class “GM” and the CSF and ventricles into the class “CSF.” The WM class was used as it is without change. What was originally left as the 10th class (i.e., unlabeled or outside) is considered as the fourth class (i.e., others). We included an illustration of an example subject in Figure 1A (top right).

### Implementation: Network Architectures

In terms of network architectures, we evaluated two state-of-the-art networks that have shown outstanding performance for different brain segmentation tasks. The first network, referred to as LiviaNET (Dolz et al., 2018b), is a single-modality 3-D fully convolutional network which was proposed in the context of subcortical brain segmentation on MRI. At the time, standard segmentation convolutional neural networks performed slice-by-slice analyses of volumetric data. Nevertheless, an important limitation of this strategy is that the 3-D context orthogonal to the 2-D axial plane was completely discarded, resulting in segmentations without 3-D consistency. To address the computational and memory requirements of 3-D convolutions, LiviaNET adopted small kernels (27^3^ voxels, Figure 1A, bounding box with green tab markers), resulting in deeper architectures with less complexity than their large-kernel counterparts. Furthermore, global and local contexts – important for both location and fine-grained details – were modeled by embedding intermediate-layer outputs in the final prediction. Figure 1B depicts the high-level architecture of LiviaNET.

The second network considered was HyperDense-Net (Dolz et al., 2018a), ranked among the top three methods in terms of performance in two different public data sets for adult (MRBRainS’13)^4^ and isointense infant brain tissue segmentation (iSeg 2017)^5^. HyperDense-Net extends the previous network, LiviaNET, by leveraging dense connectivity in the context of multimodal image segmentation. Particularly, in this network, each image modality is processed in a different path, and dense connections occur between the pairs of layers within the same path, as well as across different paths. An example of this hyperdense connectivity is shown in Figure 1C.

Network parameters of both networks were optimized via a root mean square (RMS) optimizer (Hinton et al., 2014), using cross-entropy as a cost function to measure training error. This error was tracked throughout the training process and further elaborated in Supplementary Method along with additional network initialization parameters and hyperparameters.

### Implementation: Experiment Design

There were 40 participants in total from DHCP data sets; they were split into three distinct groups: 60% of the subjects were for *training* (24 subjects), 20% were for *validation* to provide feedback on the neural network parameter tuning during training (eight subjects), and 20% were held out independently as the final *test* on the best-trained network to evaluate its generalization performance (eight subjects).

All subjects were randomly assigned to one of the three groups. The composition of the groups remains consistent throughout all experiments in both LiviaNET and HyperDense-Net network architectures.

Both networks were trained for a duration of 30 epochs composed of 20 subepoch each. At each subepoch, a total of 1,000 training subsamples (each composed of 27^3^ voxel cubes, averaging about 41 samples per subject per setting) were randomly selected and given to the network, with a batch size of two.

At the end of the 30 epochs of *training*, the best-performing model as indicated by the *validation* data sets was evaluated on the holdout *test* data set in order to report the final test Dice similarity coefficient (DSC) values.

### Implementation: Computation Platform

All training and testing were done using an Ubuntu 18.04 LTS running on a Xeon CPU E5-2600 Processor with 12 cores running at 2.0 GHz with 32 GB CPU DDR3 1,600 MHz RAM with a GeForce 1070 GPU with 8 GB of GDDR5 memory. Both HyperDense-Net and LiviaNET were implemented in Python 2.7 with Theano 1.0.0 library as per their source repositories at GitHub^6^ ^,7^.

### Performance Evaluation

The DSC was used as the metric of final performance evaluation and computed separately in GM, WM, and CSF. In the context of tissue classification problem, it is an objective measure of both correctly classifying voxels of tissue where it belongs and correctly rejecting the voxels of incorrect tissue types.

The DSC is also known as the *Sørensen–Dice* coefficient or F1 score. DSC ranges between 0 and 1 with the perfect performance scored as 1. Its derivation and references are further elaborated in Supplementary Method.

Python 3.7 stats module was used to conduct pairwise *T*-tests to compare performance metrics from the same subjects during the prediction test against ground truth across various combinations of network architecture and data. Pairwise *T*-tests were also used for inter- and intra-architectural comparisons across epochs. Bonferroni correction was applied where appropriate to ensure the family-wise error rate is constrained to below 0.05. Jupyter notebook 1.0.0 and Plotly 4.0.0 library (Plotly, Montreal, Canada) were used to plot all figures in vector format before they were touched up in Adobe Illustrator CC 2017 (Adobe Systems Incorporation, San Jose, United States) for readability and DPI compliance formatting.

## Results

### Training Performance

**FIGURE 2:**
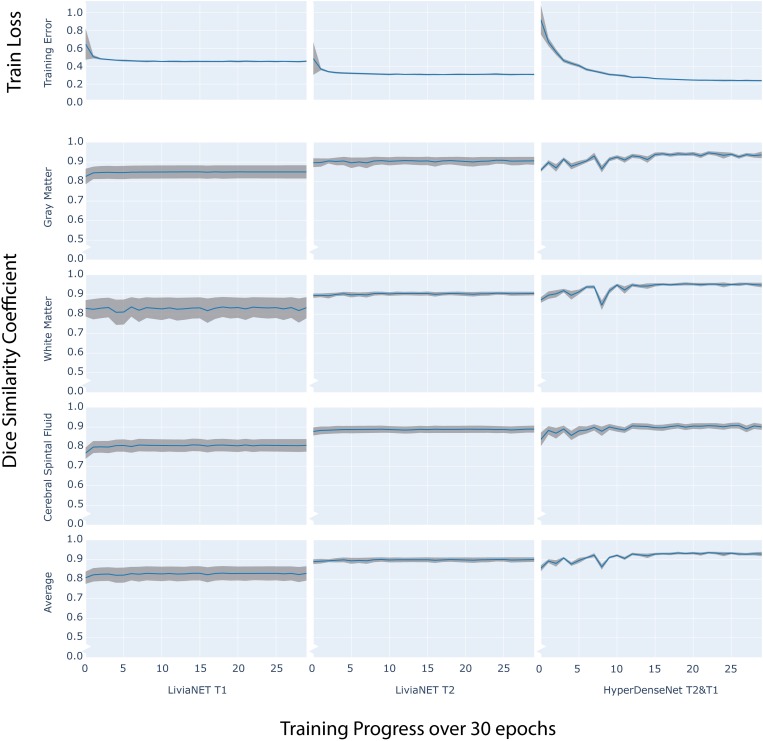
Time series plot of over 30 training epochs measuring: training loss (top row) and Dice Similarity Coefficient (DSC) of Gray Matter (Row 2), White Matter (Row 3), Cerebrospinal Fluid (CSF Row4) and Average (Row 5) across LiviaNET using T1 (Column 1), T2 (Column 2), Hyperdense-Net using both T2 and T1 (Column 3). **Blue:** Mean measure across all eight test subjects. **Gray boundary:** standard deviation across all eight test subjects.

The final model of LiviaNET using T1 achieved a stable cross-entropy cost error of about 0.47 after approximately three epochs (Figure 2, row 1, left). When undergoing the same training settings but using only the T2 acquisitions, we achieved a cross-entropy cost error of 0.33 around a similar time point, which then remained consistent until the end of the training (Figure 2, row 1, middle). The final model weights of the HyperDense-Net achieved a relatively stable cross-entropy cost error of 0.24 after almost half way into the training process and experienced a much more gradual reduction of the standard deviation of cross-entropy cost error than LiviaNET (Figure 2, row 1, right). LiviaNET T2 and HyperDense-Net appear to have demonstrated reduced standard deviation of DSC during training compared to LiviaNET T1 across tissue types (Figure 2, rows 2–5). In addition, the superimposed trace (without standard deviation for clarity) of training cost error (Supplementary Figure S2) and of average DSC (Supplementary Figure S3) over training epochs was summarized in the same chart to facilitate comparisons of performance across architectures sharing both time and performance axes.

### Test Performance

**FIGURE 3:**
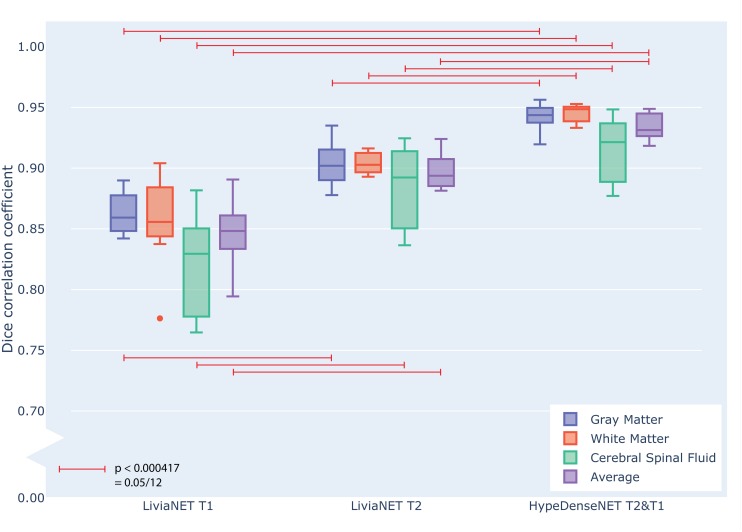
Grouped box plot showing the Dice similarity coefficient (y axis) obtained during the testing phase across eight holdout subjects for each tissue type (color) and network types (x axis groups). Horizontal red lines denote family-wise error corrected statistically significant differences measured across the DSC in the same tissue using pairwise T statistical tests.

At the end of the training, the performance of the best model was tested against previously unseen eight holdout subjects’ data as shown in grouped boxplots in Figure 3. The combination of LiviaNET and T1 data showed optimal performance at the 19th epoch and when tested resulted in prediction DSC values (mean ± standard deviation) of 0.86 ± 0.02, 0.86 ± 0.04, and 0.82 ± 0.04 for GM, WM, and CSF, respectively. Similarly, the optimal epoch for LiviaNET with T2 data was the 25th epoch and resulted in DSC values of 0.90 ± 0.02, 0.90 ± 0.01, and 0.88 ± 0.03, respectively. After accounting for multiple comparison problems via Bonferroni correction, the results demonstrate that LiviaNET using T2 data outperforms LiviaNET using T1 data significantly in most tissue types except white matter. For HyperDense-Net, the 29th epoch reported the optimal performance DSC at 0.94 ± 0.01, 0.95 ± 0.01, and 0.92 ± 0.03 for each tissue type compare to all LiviaNET results. Detailed statistical pairwise comparison results of the test performance are also included (Supplementary Table S1).

### Time Benchmark

Using the aforementioned computational platform with NVIDIA GTX1070 GPU, LiviaNET took nearly 30 h to train for T1 input data and about 31 h for T2 while requiring 8 min on average (including preprocessing time) to segment a novel neonatal brain T1 or T2 scan. On the other hand, HyperDense-Net took about 86 h to train with both T2 and T1 data. In this case, segmentation of new neonatal data set was performed in nearly 10 min (including preprocessing time).

### Visual Comparison

**FIGURE 4:**
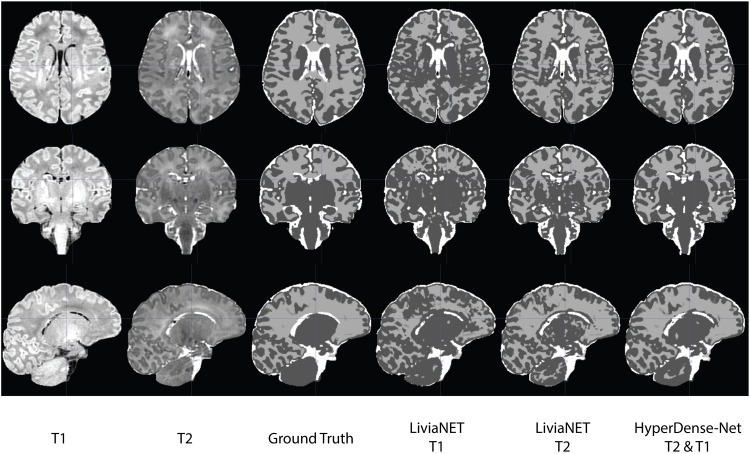
Traverse (*z* = 35), coronal (*y* = 5) and sagittal (*x* = 5) slices of input data (T1-weighted, T2-weighted and ground truth tissue segmentation) registered to the final binary segmentation output of various networks trained (LiviaNET T1, Livia NET T2, HyperDense-Net T2&T1) on a single subject from the Developing Human Connectome Project (Subject CC00379XX17). **Crosshair** set at **MNI** coordinate of [5, 5, 35] and highlights the location of the respective slides from various views.

The segmentation outputs were visually inspected for congruency and obvious mistakes. We have uploaded the eight holdout test subjects, including the preprocessed T1 and T2 volume and ground truth labels to the accompanying GitLab repositories^8^. The segmentation results as both binary classification masks and tissue probability map for each subject are available for LiviaNET T1, LiviaNET T2, and HyperDense-Net T2 and T1 weighted. Figure 4 shows a representative view of the segmentation output from one of the holdout test subjects. As illustrated, LiviaNET T1 (Figure 4, fourth column) struggled to identify WM properly especially near the deep GM regions. Across all three rows of different view perspectives, LiviaNET T1 misclassified multiple WM regions as GM, resulting the messiest view visually, congruent with its lower DSC result. On the other hand, both LiviaNET T2 and HyperDense-Net T2 and T1 segmentations resulted in better tissue separation and provided a closer match to the ground truth.

### Comparison With Previously Reported Performance

In Supplementary Table S2, the average DSCs across tissue types of the best results obtained from the present experiments, along with the ones reported in the previous implementations of it, were listed for illustrative purposes. Since only mean accuracy was reported with no standard deviation or raw results available, no statistical comparisons were made.

## Discussion

### Summary

In this current work, both LiviaNET and HyperDense-Net architectures were evaluated using the publicly available DHCP neonatal data set. We demonstrated for the first time that the dual-modality HyperDense-Net performed significantly better in the context of neonatal brain segmentation specifically across all tissue types versus the single-modality LiviaNET. In addition, LiviaNET segments the neonatal brain better with T2-weighted images than with T1-weighted images.

### Intramodel

LiviaNET has been primarily employed for single-modality inputs (i.e., T1-weighted images or T2-weighted images). Our current empirical results applying it for segmentation of neonatal T1- and T2-weighted data showed that LiviaNET with T2 contrasts performed statistically better for segmentation in neonates (Figure 3 and Supplementary Table S1). This is likely due to improved tissue contrast in neonatal T2 versus T1 and is not surprising given that neonates typically exhibit such tissue characteristics prior to the reduced contrast phase from 6 to 8 months from myelination over early development (Wang et al., 2012). This can also be observed readily in T1 and T2 raw neonatal data (Figure 4), as well as the greater high signal intensity regions observed in a simple histogram of voxel intensity plot (see Supplementary Figure S1). Lastly, visual inspection of the LiviaNET output for both T1 and T2 shows that clearly there are some deep WM which was misclassified as GM. We suspect this may be sites of early myelination (Deoni et al., 2012), resulting in altered contrasts in comparison with surrounding tissues, which resulted in misclassification.

In terms of multimodal performance, HyperDense-Net was initially envisioned as a dual-/multi-modality version of LiviaNET, which derived its name from the extensive and dense connections between the T1 and T2 streams of successive convolutional layers. In this experiment, HyperDense-Net took longer to stabilize the training error across the eight validation (not test) subjects (Figure 2, row 1, right) and had also less stable DSC which fluctuated during training (Figure 2, rows 2–5) but eventually achieved relatively stable generalizable performance (Figure 3) midway through the training. This notably stronger variation during training and validation, yet still achieving excellent generalizable results, is likely attributed to the more interconnected complexity of the architecture, requiring more observations to fine-tune the model weights through back-propagation. The observed local fluctuations in validation accuracy is a common behavior when training deep neural networks (such as those seen around epoch 8, Figure 2, rows 2 and 3, right). During training, the network parameters are updated to optimize a training objective, based on training data, which does not guarantee that the parameter updates are optimal for the validation samples. This, together with a higher learning rate at the beginning of the training, increases the chances of having these local perturbations in the validation performance, particularly in an early stage of the training. Nevertheless, as long as the validation curve converges, these fluctuations are not considered as a problem. Indeed, there exist many works, including the original HyperDense-Net (see Figure 5 in the original HyperDense-Net paper Dolz et al., 2018a), which show that these fluctuations do not hamper the network performance.

### Intermodels

All networks, regardless of design and data input type, achieved a reasonable test accuracy of higher than 80% in the independent holdout data set, and all required at least 1 day of GPU computation time to train effectively. As expected, both networks appear to benefit from the inclusion of T2-weighted images, potentially more so than from the inclusion of T1-weighted ones. This is likely due to the higher contrast found on T2-weighted images with respect to the T1-weighted ones for neonates (Supplementary Figure S1). This phenomenon is especially evident in LiviaNET-related experiments (Figure 2). Overall, the current explorative results across network architectures and data types suggest that HyperDense-Net utilizing both T2 and T1 data achieved the best statistically significant segmentation performance among all experiments (Figure 3 and Supplementary Figure S3) despite requiring a substantial amount of training time (86 vs. 30 h).

Compared to the modified LiviaNET version implemented for iSeg 2017 incorporating both T1 and T2 (Dolz et al., 2020), the current single-modality LiviaNET performance based on T2 data appears to be weaker consistently in the CSF classifications (Supplementary Table S2). Similarly, the current trained HyperDense-Net potentially performs on par or even slightly better in both GM and WM delineation while being worse in the CSF. Upon gross visual evaluation, we could not identify any major consistently common problems in the CSF relation regions, save for minor encroachment from the GM regions nearby. It might be necessary to conduct a spatial statistical parametric mapping type of analyses to truly evaluate the regions showing greater differences. However, given that we are observing this type of issues across network architectures and across data types, we suspect it might be rooted in the fundamental neonatal tissue MRI properties and should be further explored in more varied neonatal MRI acquisitions in the future.

Compared to the original HyperDense-Net training accuracy and mean DSC plot (see Figures 4, 5 from Dolz et al., 2018a), our current experiments with HyperDense-Net show similar if not slightly better and faster performance improvement from the original paper. We suspect this is also due to the improved tissue contrast at the neonatal stage versus 6-month infant data sets where onset of myelination starts to reduce the tissue contrast. Current neonatal data sets are all pre-myelination and hence may provide more information for the neural network, to better delineate tissue types, and result in faster learning and earlier observance of the performance-plateauing phenomenon. Another plausible explanation is related to the fact that for DHCP data input and ground truth, the inputs have all been preprocessed to remove non-brain-related tissues (via the Brain Extraction Tool) and to correct for non-homogeneity (N4), which could have substantially simplified the neural network’s computation effort, as the bulk of the voxels within the 3-D acquisition volumes is likely non-brain tissue.

### Performance Comparison

In terms of prediction speed, HyperDense-Net segmentation when applied to novel data was relatively fast. Although current hardware platform during the testing phase required about 8 min per participant for this study, previous reports suggest it can be even faster at 2–6 min with better-performing work-station-level graphics card such as NVIDIA Tesla P100 (Dolz et al., 2018a). Compared to other known neonatal segmentation methods such as DHCP data analysis pipeline, which takes around 7 h per participant (Makropoulos et al., 2014), or the approximately 30 min run time required by the morphological adaptive neonate tissue segmentation (MANTiS) toolbox (Beare et al., 2016), the HyperDense-Net prediction time requirement is well within reason. However, it is important to note that both of the other two traditional pipelines also conduct more granular regional identifications while both LiviaNET and HyperDense-Net are mostly tested with 3–10 classes of segmentation goals in the past, despite them being capable of conducting additional class segmentation should the ground truth be available. Since neither DHCP analyses pipeline nor MANTiS was ever officially submitted to be validated against the iSeg 2017 challenge data set, their unbiased accuracy can only be compared in neonatal data sets such as DHCP. Such comparisons, although interesting, are beyond the scope of this paper and will be the focus of our future work when we extend both neural networks to conduct more anatomical regional labeling.

### Limitation and Future Work

The fast-evolving field of computer vision has witnessed the development of many deep segmentation architectures since the seminal works such as FCN (Long et al., 2015) for the segmentation of color scenes and UNet (Ronneberger et al., 2015) for medical images. The choice of the networks analyzed in the current study is based on the competitive performance obtained in very related tasks and the public availability of their implementations. The purpose of this paper, however, is not to achieve the best performance on the task at hand but to demonstrate their reproducibility and usability for neonatal brain segmentation. We expect that this study will have a positive impact on the neuroimaging community toward the ever-widening adoption of these deep learning models in neonatal brain segmentation. Thus, future work will include more extensive evaluation of these and other state-of-the-art segmentation neural networks, to assess the neonatal brain segmentation problem. We aim to highlight efficient networks which can produce accurate and reliable segmentations while comparing them against existing traditional computer vision approaches.

In the context of comparing with the earlier works in neonatal brain segmentation, another important limitation to be considered is the limited sample size of high-quality labeled data. In the neonatal imaging world, high-quality labels coupled with high-quality medical imaging data are exceptionally rare. One of the other similar public neonatal data sets authors were aware of only consists of 10 subjects (Alexander et al., 2017). We also reviewed the subjects used in older studies in the neonatal field and found, for instance, that most of the past highly cited neonatal segmentation techniques applying traditional computer vision had tested their performance on a similar if not fewer number of subjects (Prastawa et al., 2005; Weisenfeld and Warfield, 2009). This trend persists even in more recent work as summarized in Moeskops et al. (2016, Tables 3, 4), with most studies restricted to very few subjects with no more than 20.

Regardless of sample sizes and technical solution approaches, generalization to new data is very important in the field of image segmentation, especially given the wide array of MRI contrasts possible and inter-scanner and inter-sequence variations across institutions. Current results reported are trained, validated, and tested on publicly available DHCP neonatal data, which has identical acquisition condition, scanner model, and manufacturer. Furthermore, deep-learning-based models are well known for their poor generalization capabilities on unseen data. This is particularly important in future translation of research to practice, where (1) there exists a shift between images acquired under different conditions and (2) the model needs to be retrained as these images become available. The most feasible solution to address this issue is to adopt a continual learning strategy. This approach consists on incrementally retraining deep models while avoiding any virtual loss of memory on previous seen data sets, which may not be available during retraining. This line of work will be further explored in the near future by leveraging the infrastructure of our Canadian Neonatal Brain Platform, which is currently in the progress of acquiring neonatal brain imaging data with diverse acquisition conditions from across Canada for researchers. Our final goal is to leverage such infrastructure to continuously improve the performance of networks through exposure to the ever-increasing amount of neonatal data that become available while allowing individual neonatal researchers without such infrastructures to continuously benefit from our centralized effort at retraining the neural networks to peak performance.

## Conclusion

The current study compared how two related convolutional neural network architectures addressed the automatic tissue segmentation task on neonatal brain MRIs. Among all pathways tested, HyperDense-Net showed the best performance in neonatal MRI tissue classifications. A streamlined and continuously retrained version of this will be deployed in the Canadian Neonatal Brain Platform, and we will continuously measure its performance against other competing segmentation approaches and newer network architectures.

## Data Availability Statement

The analyzed results for this study can be found in the public GitLab repository at https://gitlab.com/dyt811/M017-Results.

## Author Contributions

YD and GL conceived and designed the study. VE obtained the public database and organized it for analyses. SS prepared the ground truth for training with the help of YD. RA adapted and trained the neural networks. RA debugged the network data pipelines with the help of JO and JD. YD performed the statistical analyses, created figures and tables, and wrote the first draft of the manuscript. JD, SS, and RA wrote sections of the manuscript based on their respective areas of expertise. DL, GL, and JD provided critical feedback and organizational improvement to the manuscript. All authors contributed to the final manuscript revision and had read and approved the final submitted version.

## Conflict of Interest

The authors declare that the research was conducted in the absence of any commercial or financial relationships that could be construed as a potential conflict of interest.

## Footnotes

1 www.cnbp.ca

2 https://neobrains12.isi.uu.nl/

3 http://iseg2017.web.unc.edu/

4 https://mrbrains13.isi.uu.nl/

5 http://iseg2017.web.unc.edu/

6 https://github.com/josedolz/HyperDenseNet

7 https://github.com/josedolz/LiviaNET

8 https://gitlab.com/dyt811/M017-Results
