# Supplementary material for: Using Deep Convolutional Neural Networks for Neonatal Brain Image Segmentation
Source: Front Neurosci. 2020 Mar 26;14:207. doi: 10.3389/fnins.2020.00207 (PMC7114297; doi:10.3389/fnins.2020.00207)
Supplement: Supplementary file 1 [file Data_Sheet_1.docx]

# Supplementary Material:

## Supplementary Method

### DHCP MRI Protocol:

The data used in the DHCP cohort were collected at the Evelina Neonatal Imaging Center, London, on a 3T Philips Achieva scanner using a 32-channel phased array dedicated neonatal head coil. T2-weighted and inversion recovery T1-weighted multi-slice fast spin-echo images were acquired in sagittal and axial slice stacks with in-plane resolution 0.8mm by 0.8mm and 1.6mm thick slices overlapped by 0.8mm. T2 weighted sequence was acquired with a repetition time (TR) of 12000ms, echo time (TE) of 156ms, SENSE factor of 2.11 in the axial direction and 2.58 in the sagittal direction. T1 weighted sequence was acquired with a TR of 4795ms, inversion time of 1740ms and TE of 8.7ms along with axial SENSE factor of 2.27 and sagittal SENSE factor of 2.66.

### Dice Correlational Coefficient

which is formally mathematically defined such as in the following by Sorensen et al (1948), Dice, et al (1945): If X is the set of reference values (e.g. defined in the Ground Truth) and Y is the set of predicted values (e.g. predicted by the neural networks) for a given subject and tissue type. The DSC for each of the subject-tissue classifications is defined as:

$$DSC(X,Y)=\frac{2|X\bigcap Y|}{|X|+|Y|}$$

DSC can be intuitively interpreted as balanced measure of both sensitivity and specificity of classification performance.

### Cost Error

If we denote 𝜽 as the network parameters (i.e., convolution weights, biases and 𝛼_i_ from the parametric rectifier units), and 𝑦^𝑣^_s_ the label of voxel 𝑣 in the s-th sub-volume, the objective function (i.e., cross-entropy) becomes:

$J(\theta)$= - $\frac{1}{S\cdot V}\sum_{s=1}^{S} \sum_{v=1}^{V} \sum_{c=1}^{C} \delta\left( y_{s}^{v}=c \right)\cdot\log p_{c}^{v}\left( x_{s} \right)$

where p^𝑣^_c_ (x_s_) represents the softmax output of the network, which represent the predicted probability maps per class c and at each voxel v, when the input sub-volume is x_s_.

### Network Initialization:

To initialize the network parameters we resort to the approach proposed in the original LiviaNET and HyperDense-Net works, which was originally proposed in (He et al., 2015) for fast convergence of very deep neural networks. This strategy uses a zero-mean Gaussian distribution of standard deviation $\sqrt{2/\eta_{l}}$ to initialize the weights on layer $l$, where $\eta_{l}$represents the number of connections to units in that layer.

## Supplementary Figure


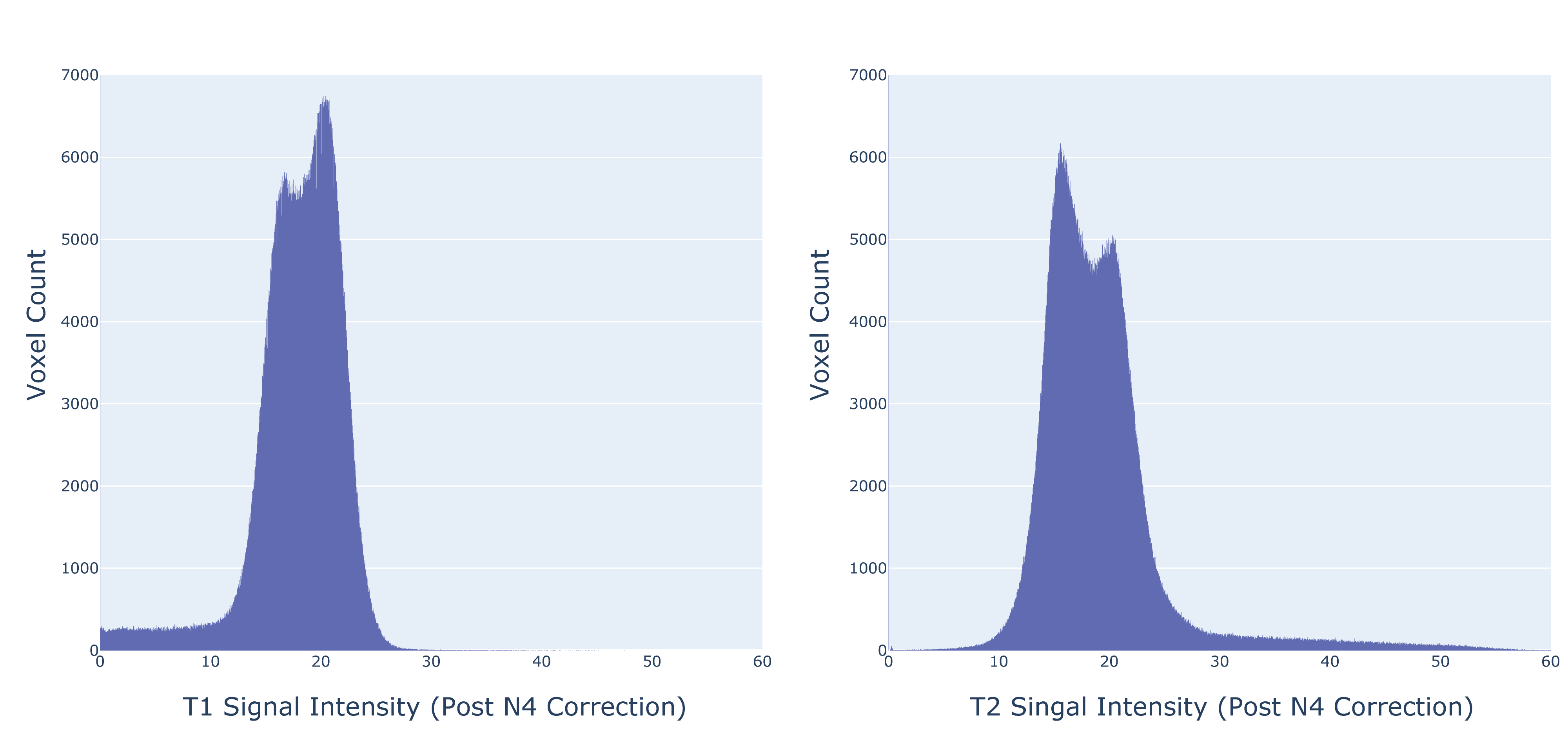


### Supplementary Figure S1: Histogram plots of the preprocessed T1 (left) and T2 (right) weighted MRI voxel signal intensity data in DHCP Subject Number CC00379XX17_ses-120400.

Note that T2 had noticeable longer tail at high signal intensity area vs T1.


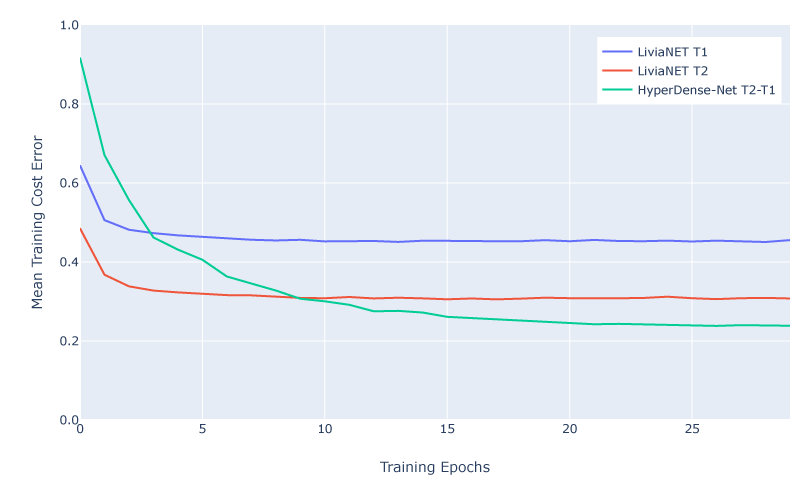


### Supplementary Figure S2: Time series plot of training cost error of all network architecture tested over 30 epochs.


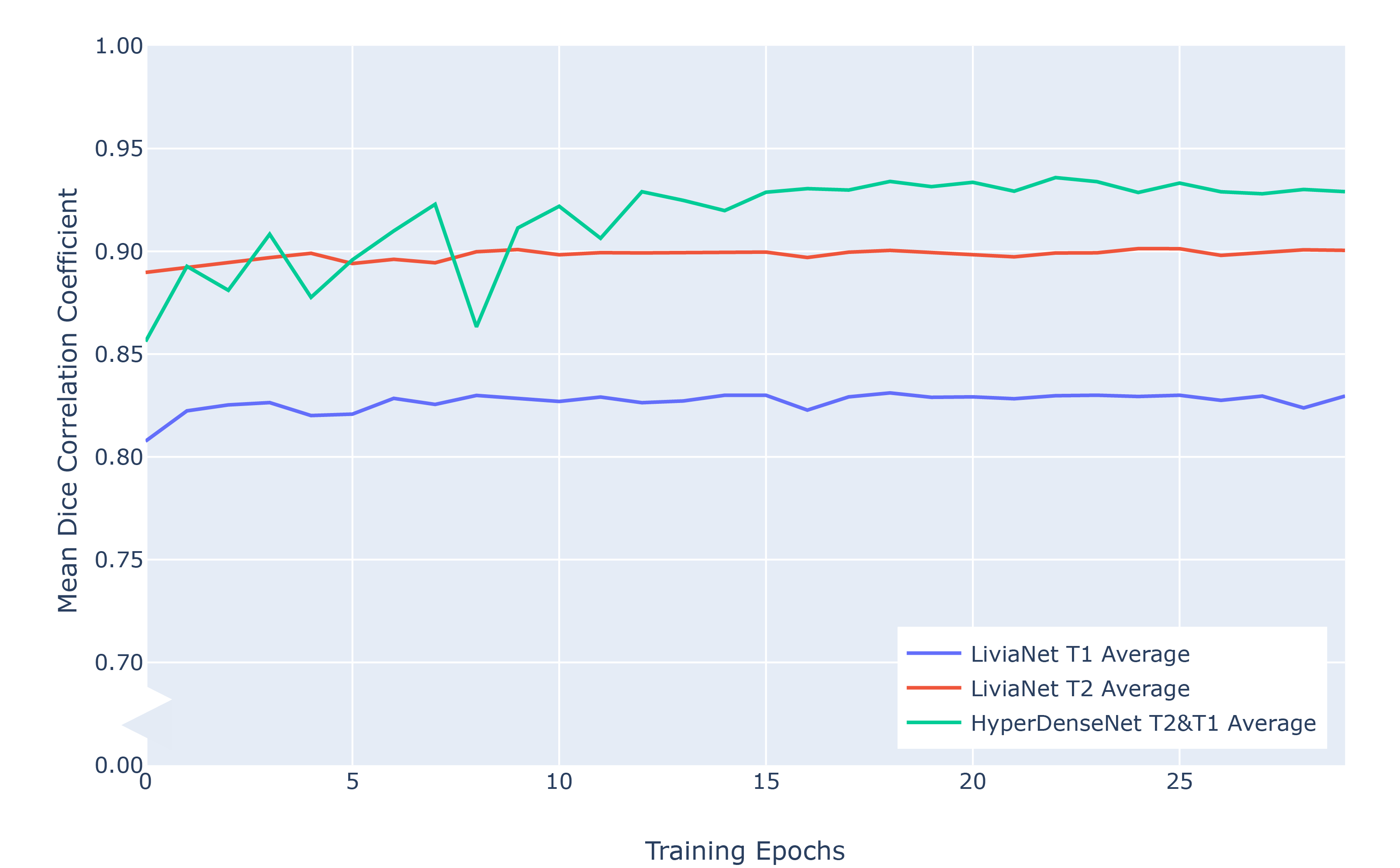


### Supplementary Figure S3: Time series of Dice Similarity Coefficient measurement of average tissue classification of all network architectures tested.

## Supplementary Tables


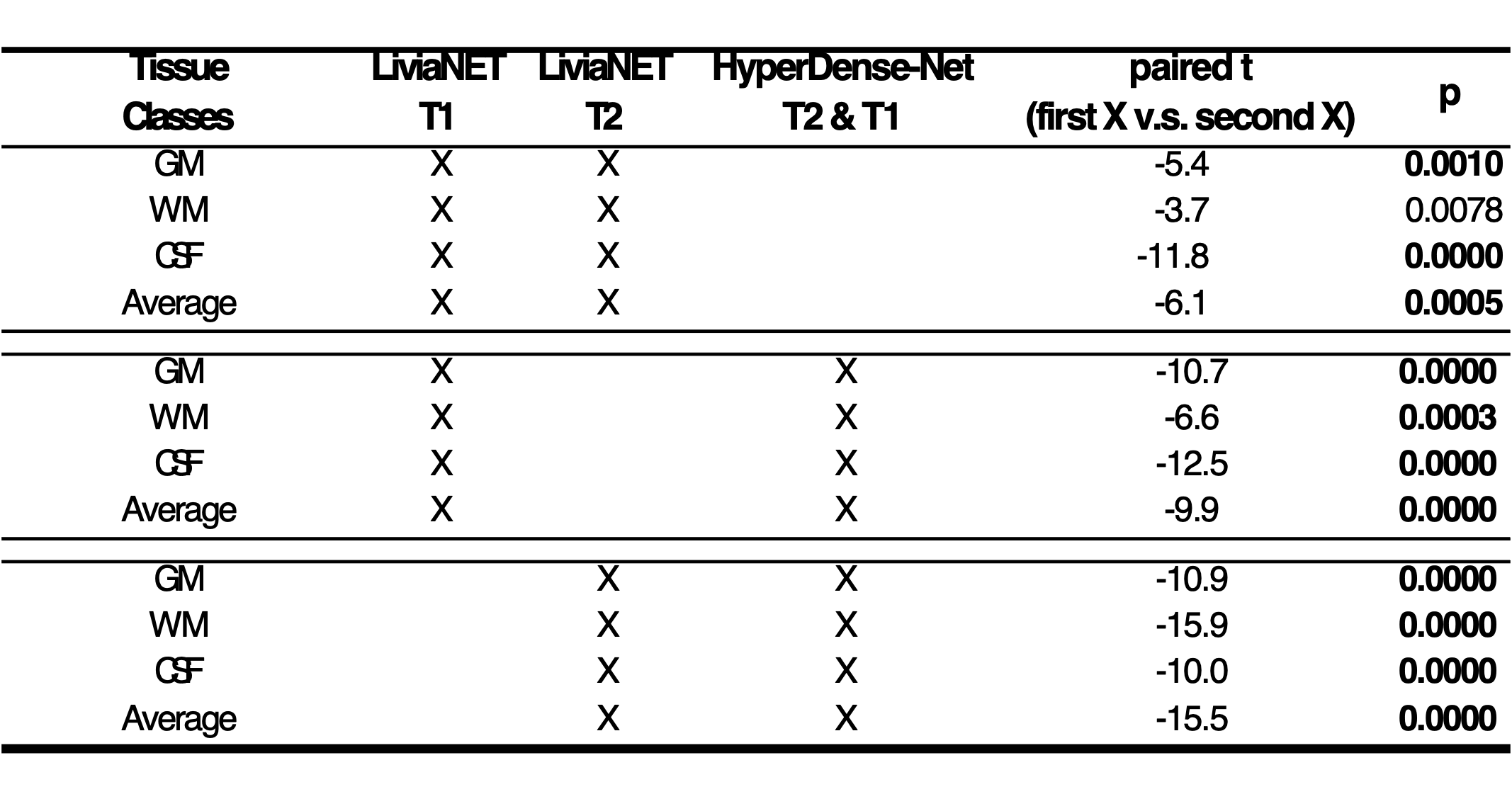


### Supplementary Table 1: Pairwise comparison of Dice similarity coefficient of all eight test subjects between different combinations of data across network architectures. Bold indicates statistically significant results after Bonferroni Family Wise Error correction at p < 0.0042 = 0.05 / 12.

### Supplementary Table 2: Mean DSCs obtained during the testing phase for each tissue type for current study (Bold) vs previously reported performance in other competitions.

## Supplementary Discussion

### Training Error Rate

LiviaNET for both T1 and T2-weighted images respectively achieved a stable training error rate after five epochs (**Figure 2, Row 1 left and middle**) while showing fairly steady DSC performance (**Figure 2, Row 2-5 left and middle**), thus suggesting perhaps early stopping of the training is feasible and full 30 epochs may not be entirely required. Curiously, the extensive additional training actually did not introduce model overfit as shown on the test set (**Figure 3**) and in fact mirror the DSC values during training and validation **(Figure 2, Row 2-5 left and middle)**. During the early training phase, unimodal LiviaNET typically show a quick reduction of the training error far sooner than HyperDense-Net (**Figure 2 Row 1 left and middle**), suggesting the unimodal LiviaNET weights potentially stabilizing quickly at the beginning of the training but plateaued quickly around fifth epochs in terms of performance with no subsequent improvement (**Supplementary Figure S3**). Whereas multimodal network training error distribution never truly decreased much (**Figure 2 right**) and learned at a slower rate and slowly flattens out continuously (**Figure 2 right column,** **Supplementary Figure S3**). We postulate that the slower convergence is most likely related to the increased complexity of the HyperDense-Net which has more than four times the model weight parameters versus LiviaNET. This makes that even though HyperDense-Net has a higher representation power, it requires more gradient steps to successfully disentangle the useful information (e.g. T2 contrasts) from the less useful information (e.g. T1 contrasts) and converge to its highest performance.

### Early Stopping and Further Training Time Reduction

Early stopping of training had previously been shown to be an effective strategy to improve performance (Yao et al., 2007). With LiviaNET training time at almost half of the HyperDense-Net and given how error rate (**Figure 2, Row 1 left and middle**) and DSC stabilized early in the training (**Figure 2, Row 1 left and middle**), the training time can be potentially be even further reduced via early stopping to as low as maybe five hours on a NVIDIA GTX 1070 consumer level graphics card. This training time accuracy trade off may be desirable for future faster iterative exploration of some datasets instead of waiting four days to see the results that may differ by around 5% DSC values.

## Supplementary Material References:

Dice, L.R. (1945). Measures of the amount of ecologic association between species. *Ecology* 26**,** 297-302.

He, K., Zhang, X., Ren, S., and Sun, J. (Year). "Delving deep into rectifiers: Surpassing human-level performance on imagenet classification", in: *Proceedings of the IEEE international conference on computer vision*), 1026-1034.

Sørensen, T.J. (1948). *A method of establishing groups of equal amplitude in plant sociology based on similarity of species content and its application to analyses of the vegetation on Danish commons.* I kommission hos E. Munksgaard.

Yao, Y., Rosasco, L., and Caponnetto, A. (2007). On early stopping in gradient descent learning. *Constructive Approximation* 26**,** 289-315.
